# Sounds of Silence in Times of COVID-19: Distress and Loss of Cardiac Coherence in People With Misophonia Caused by Real, Imagined or Evoked Triggering Sounds

**DOI:** 10.3389/fpsyt.2021.638949

**Published:** 2021-06-30

**Authors:** Antonia Ferrer-Torres, Lydia Giménez-Llort

**Affiliations:** ^1^Centro Médico Psicológico L'Alfatier, Barcelona, Spain; ^2^Department of Psychiatry and Forensic Medicine, Faculty of Medicine, Universitat Autònoma de Barcelona, Barcelona, Spain; ^3^Institut de Neurociències, Universitat Autònoma de Barcelona, Barcelona, Spain

**Keywords:** misophonia, distress, loss, cardiac coherence, heart rate variability, COVID-19, confinement

## Abstract

The extreme, unprecedented situations in the current COVID-19 pandemic are risk factors for psychosocial stress for the entire population. However, strict confinement had a particular impact on people suffering from misophonia and their families. Misophonia is a condition in which hearing certain sounds triggers intense anger, disgust and even severe autonomic nervous system responses. This prospective cohort study examined the impact of strict confinement (Spain, March 14–June 21, 2020) on a sample of 24 people (16 women and eight men) who had been diagnosed with moderate to extreme misophonia and were regularly attending a medical psychology center in Barcelona. The 3-month period of confinement caused general emotional maladjustment, distress, and a transitory crisis. Long-term biomonitoring of their heart variability rate (HRV) enabled to identify a significant increase in physiological arousal after the confinement period, which had already been recorded in a loss of cardiac coherence under basal rest/relaxation conditions. Certain auditory stimuli triggered adverse responses, lowered HRV scores, and an increased stress level and heart rate. Loss of cardiac coherence in their responses to these auditory stimuli (triggering mouth, nose and other sounds), as well as to non-triggering mouth, nose and other sounds was increased when compared to two assessments performed during the previous year. Despite the limited sample size, sex differences were observed in the incidence. Loss of cardiac coherence worsened with the severity of the misophonia. Most importantly, imagined or evoked triggering sounds, as well as real ones, were enough to cause the aversive responses, as displayed by the increased loss of cardiac coherence with respect to the at-rest basal level. A semi-structured interview revealed the exceptional nature of the situations, increased hyper-sensorial sensitivity, fear of being infected with or dying from COVID-19, the patients' coping strategies, and the difficulties and constraints they faced. Finally, the article gives recommendations for better management of misophonia. Improved knowledge of this disorder would help address the current lack of health and social care, hopefully preventing this shortfall's impact on social and affective relationships, which are particulary important for well-being now and in the coming periods of physical distancing measures.

## Introduction

### COVID-19, Confinement and Secondary Impact of COVID-19 Pandemic

Since the WHO declared the COVID-19 outbreak a pandemic on March 11, 2020 (1) an increasing number of scientific reports have documented the psychological affects upon the population. These have been attributed to confinement, as well as the direct and indirect effects of the illness itself (2, 3). Added to this are the loss of loved ones and associated grieving processes, as well as the impacts of a sudden health and socioeconomic crisis of unprecedented magnitude.

Even before the COVID-19 pandemic, studies warned that confinement responding to outbreaks of viral diseases such as SARS and H1N1 flu produced negative psychological effects (3). However, Spain had no choice but to implement strict confinement measures to put a halt to the dramatic spread of the disease through its population and to prevent its health system reaching saturation point. In Spain, a strict lockdown of 47 million people was put into place from March 14 to June 21, 2020, and limited the free movement of citizens to essential acts such as the acquisition of food and medicine or going to medical centers or the workplace, resulting in the confinement of the population in their places of residence. The emergence of anxiety, depression, insomnia, denial, anguish and fear was particularly common, as was the aggravation of existing conditions (4). The Spanish scientific-technical guidelines for managing COVID-19 included a section that detailed how the pandemic had contributed to the mental health crisis. The document paid particular attention to health professionals and hospitalized patients, people with pre-existing mental illness, and individuals in difficult situations caused by isolation and the economic crisis. The international scientific community has described this phenomenon as “the untold toll” – the effects of the pandemic on patients without COVID-19 (5) and can be also referred as secondary impact of COVID-19 pandemic.

### Misophonia and Co-morbid Disorders

At present, misophonia is not included within the International Classification of Diseases (ICD-11) and does not appear in the Diagnostic and Statistical Manual of Mental Disorders (DSM-5). However, several studies have indicated that misophonia is a disorder associated with a wide variety of psychiatric symptoms (6–9), including anxiety that acts as a channel for anger (10, 11). Furthermore, according to studies by Schröder et al. (12), people with misophonia report suffering a significantly higher percentage of anxiety symptoms than control groups. Another recurrent disorder in the misophonic population is depression (13), which was diagnosed in 22% of a sample of 50 people suffering from misophonia (14) and in 9.61% of a sample of 52 (8). Both studies revealed a positive correlation between depression and the severity of misophonia symptoms. Rouw and Erfanian (15) also found that post-traumatic stress disorder (PSTD) was one of the most common diagnoses in people with misophonia.

### Mapping Neuronal Basis of Misophonia

Neuroimaging research has revealed that there might be a neural basis to misophonia (16, 17). Individuals with misophonia have been shown to have greater activity in the anterior insular cortex in response to signals that trigger misophonia than in response to non-triggering or universally unpleasant signals, compared to the control group (18). The existence of greater functional connectivity in the misophonic group between the anterior insular cortex and the ventromedial prefrontal cortex, posteromedial cortex, hippocampus, and amygdala indicates that there are neuronal bases for the heightened, emotional response to triggering stimuli. Schröder et al. (12) demonstrated networks' prominent involvement in misophonia and that the right anterior cingulate cortex and the right insula are activated to a greater extent in people with the condition (12).

### HRV and Cardiac Coherence as Objective Physiological Measures of Misophonia

At a physiological level, people with misophonia show higher skin conductance responses to auditory stimuli that are triggering for them, compared to healthy controls (19). This increase in conductance was thought to reflect an autonomous physical component in the misophonic reaction. Also, heart rate variability (HRV) describing the physiological phenomenon of variation in heartrate in the beat-to-beat time interval between consecutive heartbeats (20–25) is considered an effective instrument that is able to quantitatively assess the response of the autonomic nervous system (ANS) to certain situations (26). HRV analysis is therefore recognized in clinical settings as a powerful indicator of the relationship between psychological and physiological processes (27). As some scholars have pointed out, HRV is accepted to be a cost-effective method with a high level of validity and reliability, regardless of any cardiovascular disease the patient may have (28). It has been used in several studies to investigate the reactivity of the ANS to sounds (29, 30). Also, a number of interventions aimed to improve health and wellbeing though the adjustment of ANS balance train patients to achieve “cardiac coherence,” the coupling and synchronization of the rhythm of breathing to the rhythm of the heart [i.e., (21, 31–35)].

### Misophonia Emotional, Behavioral, Physical, and Physiological Components

When exposed to auditory stimuli, which may also be termed “triggering sounds,” people who suffer from misophonia experience intense and unpleasant emotional, behavioral, physical and physiological reactions. Thus, responses to triggering sounds or “misophonic responses” are usually: emotional (anxiety, anger, disgust, extreme irritation, the feeling of losing emotional control, the feeling of being overloaded by stimuli); behavioral (feeling the need to flee or escape from the place where the sounds are being produced); physical (pressure in the chest); or physiological (increased heart rate), in addition to many others (19, 36, 37). The most common somatic reactions in these patients are pressure in the chest, tension in the arms, head, or the whole body, and increased heart rate, increased body temperature, physical pain, or breathing difficulties (19). People with misophonia tend to avoid social situations or anticipate triggering stimuli, which leads to a deterioration of their social and functional life. Characterizing the complex neural underpinnings of misophonia and the interplay of neural systems (38) to develop the behavioral, cognitive and emotional responses to misophonic triggers is among the key aspects considered in the road map for advancing in the scientific research agenda in this new field (36).

### Misophonia Common Triggers

Sounds such as chewing, lip-smacking or breathing are among those that most frequently provoke intense anger and physical arousal (19). According to Jager et al. (39), the most disturbing sounds are eating (96%), followed by nasal or respiratory sounds (85%), these being the two key groups used to diagnose misophonia. In addition to these two groups, other triggering stimuli, such as sounds made by other humans, animals, objects, and the environment can be added. Concurrently, visual or movement triggers are also noted.

### Misophonia and Secondary Impact of COVID-19 Pandemic

Jastreboff first described this phenomenon in 2001 (40). Since then, scientific studies of misophonia, while still scarce, have increased in number [i.e., (14, 18, 19, 36, 39, 41–44)]. Misophonia has only been discovered recently, yet it is a fairly common condition. A study conducted with college psychology students reported that 19.9% had clinically significant misophonia symptoms (10). Further work research of this research team found 6% incidence among Chinese university students. Besides, their work provided further evidence on misophonia symptoms being associated with noteworthy impairment, as well as general sensory sensitivities, obsessive-compulsive, anxiety, and depressive symptoms (43). During confinement, interpersonal and social conflicts have also increased (2, 45–47). Factors such as the reorganization of domestic space, the intrusion of virtual spaces into family life and multitasking, have made the home into a space in which activities take place that were previously conducted outside of it. Additionally, the crisis situation has made it impossible for families and individuals to go through necessary adaptation processes, generating intra-family and social stress. Due to a lack of knowledge and information, misophonic patients have not been taken into account at the socio-sanitary or legal levels. This lack of assistance results from the fact that the disorder is unfamiliar to a significant number of psychological health professionals and a large proportion of the population: even those who suffer from it.

### Current Study on the Secondary Impact of Strict Confinement During COVID-19 Pandemic on Patients With Misophonia

The objective of this prospective study was to determine the impact of strict confinement on the HRV of misophonic patients in Spain, using a longitudinal design. For that purpose, a pre-post analysis of the HRV assessment was conducted in people diagnosed with misophonia who were regularly attending a medical psychology center in Barcelona. The study hypothesized that, during confinement, the following HRV-related changes would be observed: [1] there would be a worsening in responsiveness to auditory triggers, with respect to two preceding basal periods; [2] a stressful period of confinement would increase heart rate variability in patients with misophonia, and [3] not only real but imagined or evoked triggers would result in a loss of cardiac coherence. Finally [4], it was hypothesized that the semi-structured interviews on their fears (the fear or being infected or dying), relationships and emotional states, in conjunction with their self-assessments, would confirm the increased impact or perception of stressful events, while also revealing their coping strategies.

## Materials and Methods

### Participants

The prospective study started on June 29th, 2019 with a cohort of 24 individuals (16 women and eight men), who were regular patients at a medical psychology center in the city of Barcelona. None of them had initially requested a specific consultation for treatment for misophonia. These patients were later diagnosed with misophonia at the center using a recent scale: Amsterdam Misophonia Scale [A-MISO-S, Schröder et al. (41)].

Patients first came to the center in the belief that they had other conditions. In fact, 99% (one woman/24 patients) of them were completely unaware that they were suffering from misophonia. After initial diagnosis and subsequent check-ups, we tested these regular patients for misophonia. The cases identified as misophonic after June 2019 were selected for this study. The inclusion criteria were as follows: Over 18 years of age; Having agreed to participate in the study; Having spent the national strict confinement period at home, following it according to the rules; Being a regular patient of the center; Having been diagnosed with misophonia after June 2019; At least mouth and nasal sounds having been identified as triggering stimuli in the patient's diagnosis; Having a minimum of four triggering stimuli.

Exclusion Criteria were: History of phonophobia, hyperacusis, tinnitus, tympanic membrane perforation or temporomandibular disorders (TMDs); Having any degree of hearing impairment, from mild to severe, following the Bureau International d‘Audio-Phonologie (BIAP) criteria, which indicates mild hearing loss starting at 20dB; Have received a treatment for misophonia.

## Evaluation Tools

### Diagnosis of Misophonia

During the diagnostic phase, the Amsterdam Misophonia Scale (A-MISO-S) was used to assess whether patients were suffering from misophonia (41). For the only purpose of this study, in order to facilitate data collection and analysis, the five levels of this numerical scale (41) were labeled as follows: level 0, not misophonic (for a score of 0–4, subclinical); level 1 (5–9, mild); level 2 (10–14, moderate); level 3 (15–19, severe); level 4 (20–24, extreme).

### Physiological Measurements

**Figure 1:**
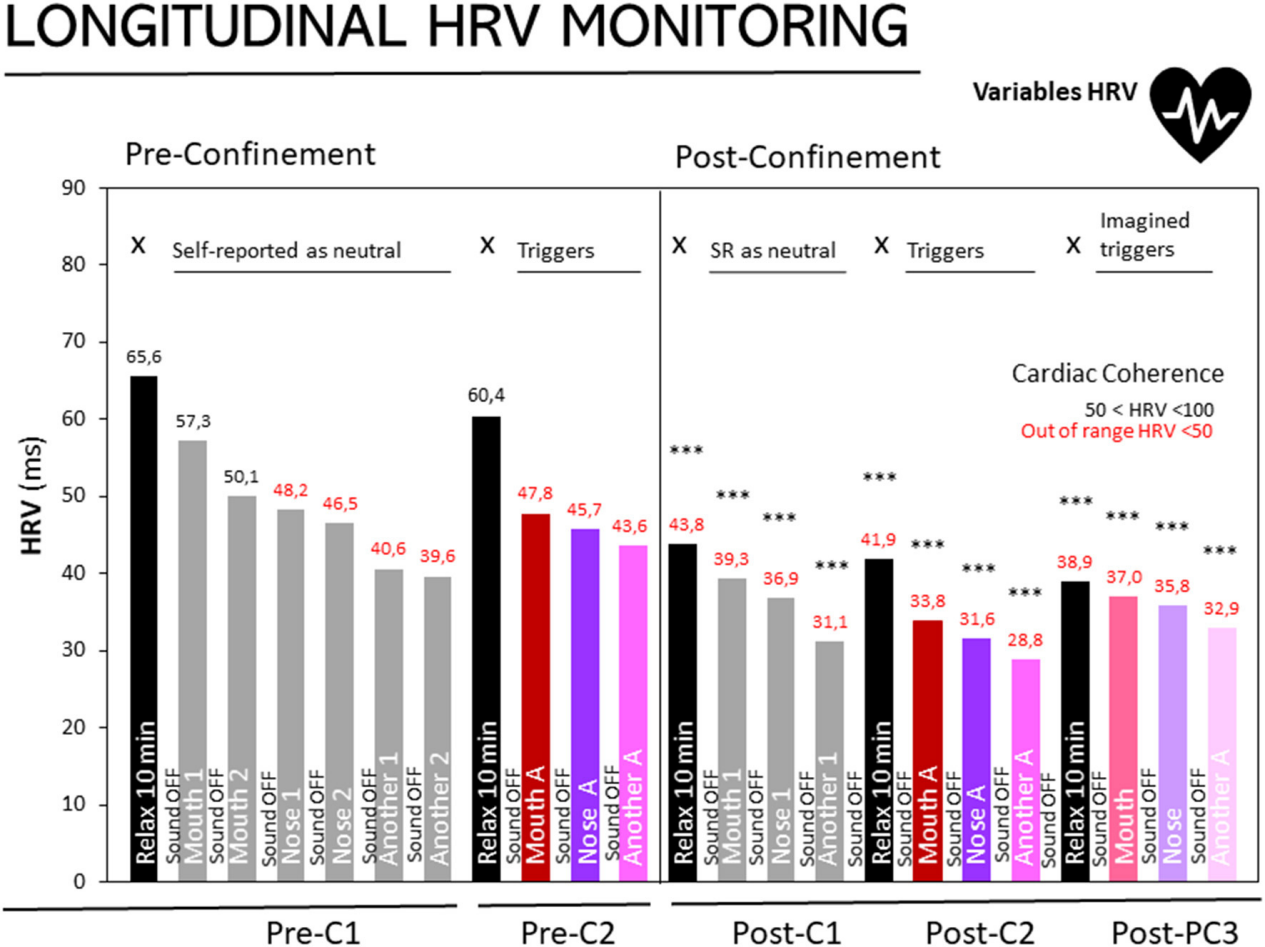
Longitudinal monitoring of HRV in people with misophonia at three different stages. Results are expressed as medians of HRV (ms). The 25, 50, and 75th percentiles are detailed in the Supplementary Table in Supplementary Material Pre-confinement- First assessment (Pre-C1), Pre-confinement Second Assessment (Pre-C2), Post-confinement First (Post-C1), Second (Post-C2), and Third (Post-C3) Assessments. Sound on and sound off as depicted inside and between the bars, respectively. Black bars: HRV during the 10 min of relaxation, X: without sound stimuli. Gray bars: Sounds self-reported (SR) as neutral: Mouth sound 1, mouth sound 2, nose sound 1, nose sound 2, another sound 1, another sound 2. Trigger sounds: Magenta bars, mouth sound A; Purple: nose sound A, Pink: another sound A. Imagined sounds: Soft Magenta bars, imagined mouth sound A; Soft purple, imagined nose sound A; soft pink, imagined nose sound A. Normal cardiac coherence (50 < HRV < 100); in red, cardiac coherence out of range (<50). Statistics: ***p < 0.0001 Wilcoxon's rank-sum test, as compared to “Basal level, Relaxation 10 min” of the first assessment.

In order to measure the impact that confinement, the degree of balance-imbalance of the ANS was assessed. The patient's psychophysiological response was recorded by measuring HRV (ms) during exposure to certain auditory stimuli and in a state of rest or relaxation. The emWave® technology (emWave® Desktop and emWave® PSR; HeartMath Institute, California, USA), a software that allows heart behavior patterns to be observed and measured on a computer, tablet, or cell-phone screen in real time, was used. The software is a heart-rate coherence trainer, designed to assess, prevent, control and reverse the effects of stress. Figure 1 provides an example of an HRV read-out upon exposure to a sound, the results remaining without the normal range (48). As indicated, each of the sounds was evaluated through time domain analysis, using a statistical method sourced from the Task Force of The European Society of Cardiology and The North American Society of Pacing and Electrophysiology (49). This is currently one of the most widely used methods.

### Procedure

The protocol was designed to assess HRV in three different stages: [1] basal level – after obtaining the misophonia diagnosis (2019); [2] second assessment for a retest- after completing the first half of the program, and [3] post-confinement - at the end of June 2021 after having been through the period of confinement responding to the COVID-19 pandemic (March 14 to June 21, 2020).

On each day of assessment, the participants were asked to have had a light meal in the two hours before the test, but not to have ingested alcohol, coffee, tea, chocolate or any other stimulants. They were informed about the duration of the study, what it was about, and the number of sounds they would be exposed to, but at no stage did they know what auditory stimulus would be presented to them. The assessments were conducted in a soundproof room, with dim light. During assessment sessions, no interruptions were allowed and the patient was physically isolated from the rest of the center.

In the three assessments, prior to the exposure to the sounds, the patient was asked to perform a relaxation exercise for 10 min, followed by sound exposure for 5 min. Between each period of sound exposure, during the intertrial time of 5 min, a rest/ relaxation was again performed.

The exposures were as follows:

Pre-confinement First assessment (Pre-C1) – The patient was exposed to non-triggering sounds (understood as those sounds the participant had not identified as triggering misophonia at the time of diagnosis), two sounds related to eating, two nasal sounds and two other sounds. All these sounds had been randomly selected by the psychologist administering the test.
Pre-confinement Second assessment (Pre-C2) – The patient was exposed to a total of three sounds they had identified as triggering misophonia: one chewing food, one nasal, and one miscellaneous. These sounds had been selected in advance by the psychologist administering the test. In this assessment, the sounds were taken from each participant's Personalized Sound Bank (PSB). The procedure for creating the PSB is explained at the end of this section. A total of 15 sound banks were used.
Post-confinement (PC) – Consisted in three assessments: (Post-C1) exposure to non-triggering sounds, in which the procedure completed in the first assessment was repeated, but using only three sounds identified as non-triggering – 1 chewing food, 1 nasal and 1 miscellaneous; (Post-C2) exposure to second assessment triggering sounds, the second assessment being repeated in the same way; and (Post-C3) imagined triggering sounds used in the second assessment, in which the patient was asked to imagine or recall the three triggering sounds used in the preceding assessment of this stage. There was no sound exposure during this assessment.

### The Personalized Sound Bank (PSB)

The PSB consisted of a personalized sound file for each patient and it was set up by a sound technician using a sound meter, a computer, headphones, the list of triggering sounds mentioned by the patient, and the interview notes.

PSBs were created as follows: Patients had to record the situations and stimuli that triggered misophonia in their daily life for 1 month. This record had to be brief, and the patient had to rate the trigger of misophonia from 0 to 10, differentiating their physical reaction from the emotional one. At the end of the month of recording, an interview was conducted in which the patient handed over and explained the list of situations. From this list, the sounds corresponding to the patient's description in the interview were selected from the 15 sound banks.

Three alternative sounds were recorded that matched the patient's description. A pre-assessment of triggering sounds was carried out, where patients were asked to confirm whether the selected sounds resembled those they had described by them in their month-long record. After exposure to the sound, the patient had to choose one of the following options:

[1] It does not resemble the recorded sound at all; [2] It somewhat resembles the recorded sound; [3] Yes, this is the sound; [4] This is the exact sound situation experienced. The sound was only accepted when the patient validated it with answer 3 or 4. If the patient agreed, the sound was recorded as definitive. If the patient rejected the sound, it was canceled and a new one was presented for the same task. For each situation, three alternative sounds had been filed, using identical triggers described by the patient. All the sounds were normalized so that they did not exceed 70dB, as measured with a sound meter. PBS were stored in a computer, from where they were used as outputs for the second assessment of the post-confinement stage.

### Self-Reporting

After the confinement period and having conducted the third and final HRV assessment (post-confinement stage), a semi-structured interview was carried out with each patient. Factors related to interpersonal relationships, emotions and other areas connected to misophonia were investigated by conducting a semi-structured interview, as detailed in **Table 2**. The descriptive analysis determined patients' sociodemographic characteristics and the reasons why they believed they had modified their behavior during confinement.

### Data and Statistical Analysis

The statistical analysis derived from the HeartMath tool was performed using IBM SPSS-software (version 25), through which both descriptive and inferential procedures were carried out. Statistical analysis of HRV was carried out by means of Wilcoxon signed rank tests for non-parametric statistical analysis since the variables did not follow a normal distribution and there was no homogeneity of variances.

## Results

### Demographics

The demographics of the sample of 24 participants, with a sex ratio of 2:1 women to men, are depicted in Table 1.

**Table 1:** Demographics and misophonia diagnoses.

	**Total**	**Women**	**Men**
	**(*n* = 24)**	**(*n* = 16)**	**(*n* = 8)**
Age (years)	45.75 ± 11.12	47.63 ± 11.83	42.0 ± 9.07
Marital status			
Married	11	8	3
Single	7	4	3
Divorcee	4	3	1
Widow	1	1	0
Level of education			
Primary	0	0	0
High school	12	6	6
University	12	10	2
Misophonia (M+)			
Level 2, moderate (M+ 10–14)	10	6	4
Level 3, severe (M+ 15–19)	10	7	3
Level 4, extreme (M+ 20–24)	4	3	1

*Assessed using the Amsterdam Misophonia Scale (A-MISO-S, 27). The levels were adapted by the authors for the only purpose to facilitate analysis in the present work*.

### Longitudinal Monitoring of HRV

Figure 1 shows the results of participants diagnosed with misophonia in the three stages: basal level, retest and post-confinement assessments. For the basal level and retest assessments, the HRV measurements in a state of relaxation (black bars) produced a score within the normal range (>50). In contrast, during the post-confinement assessment, the three scores for the relaxation period (black bar at PC1, PC2, and PC3) progressively decreased. Significantly, in all the post-confinement assessments, including those referring to the 10 min of relaxation, the scores were HRV < 50, outside the normality range (Wilcoxon rank test, *p* < 0.001 *vs*. basal level).

### HRV According to the Different Levels of Misophonia

#### Second Assessment

**Figure 3** depicts HRV responses to three non-triggering auditory stimuli at the second assessment, according to participants' level of misophonia (moderate, severe or extreme), as diagnosed using the A-MISO-S test. A negative relationship can be observed between the degree of misophonia and the results obtained by exposure to non-triggering sounds: the higher the degree of misophonia, the lower the HRV score (misophonia-2: 20.32; misophonia-3: 12.0; misophonia-4: 10.5). The same relationship also appears in the relaxation or resting stage assessments: in this state of rest or relaxation, the HRV value progressively decreases, the more severe the misophonia.

#### Post-confinement

**Figure 2:**
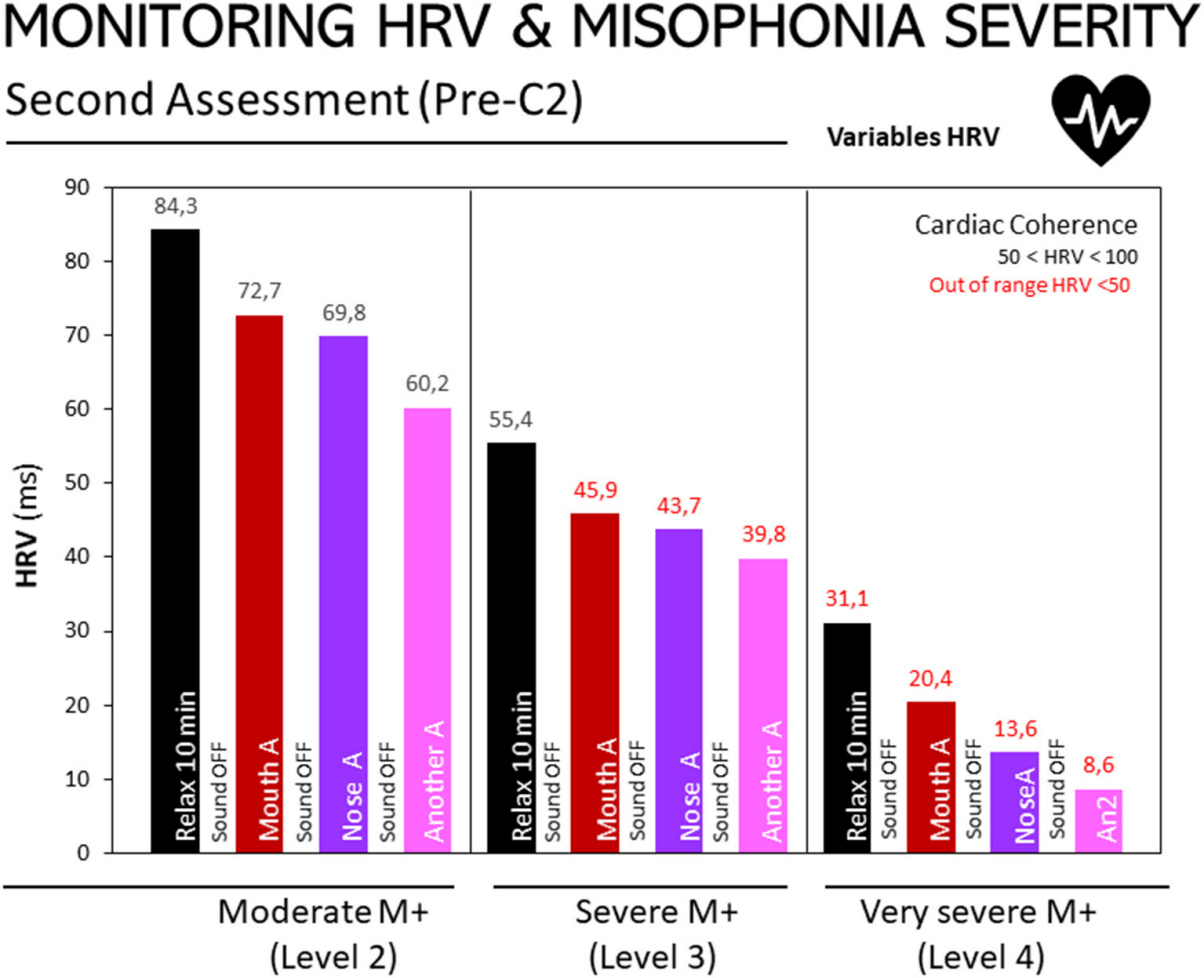
Monitoring of HRV in people with misophonia during the second assessment, according to the severity of the disorder. Results are expressed as medians of HRV (ms). The 25, 50, and 75th percentiles are detailed in the Supplementary Table in Supplementary Material. Bar codes as in Figure 1. Moderate M+ (Level 2, *n* = 10), severe M+ (Level 3, *n* = 10), and extreme M+ (Level 4, *n* = 4) exposed to non-triggering auditory stimuli (mouth, nose, other). Misophonia was assessed using the Amsterdam Misophonia Scale [A-MISO-S, (41)] and the levels of misophonia were adapted by the authors for the only purpose to facilitate analysis in the present work.

Figure 2 illustrates HRV responses at the post-confinement stage and according to participants' level of misophonia, as diagnosed using the A-MISO-S test. The responses are shown for non-triggering auditory stimuli, the triggering auditory stimuli, and the imagined/evoked stimuli.

**Figure 3:**
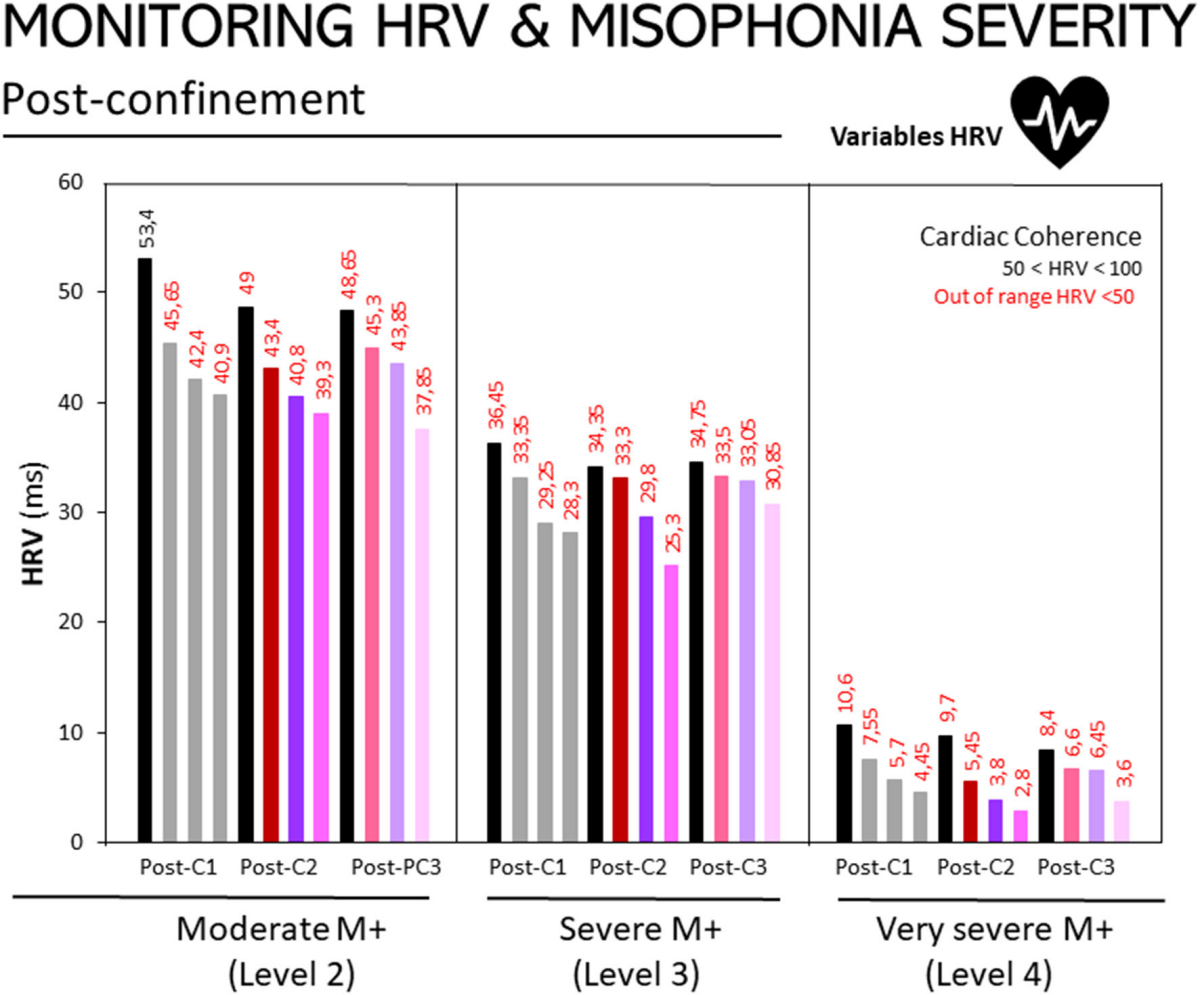
Monitoring of HRV in people with misophonia during the three assessments conducted at the post-confinement stage, according to the disorder's severity. Results are expressed as medians of HRV (ms). The 25, 50, and 75th percentiles are detailed in the Supplementary Table in Supplementary Material. Bar codes as in Figure 1. Moderate M+ (Level 2, *n* = 10), severe M+ (Level 3, *n* = 10) and extreme M+ (Level 4, *n* = 4) exposed to non-triggering auditory stimuli (mouth, nose other), triggering auditory stimuli (mouth, nasal, other), and imagined/evoked triggers (mouth, nose, other). Misophonia was assessed using the Amsterdam Misophonia Scale [A-MISO-S, (41)] and the levels of misophonia were adapted by the authors for the only purpose to facilitate analysis in the present work.

The same trend as in Figure 3 can be observed – the higher the degree of misophonia, the lower the HRV score, as shown in the following figures. Assessment 1: M+ [2], 132.66; M+ [3], 85.94; M+ [4], 16.98. Assessment 2: M+ [2], 117.91; M+ [3], 79.44; M+ [4], 11.78. Assessment 3: M+ [2], 124.64; M+ [3], 87.06; M+ [4], 16.05.

#### Self-Reporting

Table 2 shows the self-reported results, constructed on the basis of a semi-structured interview carried out with the patients after confinement. It includes their own evaluation of their interpersonal relationships and their ideas about why their emotional and relational states worsened. This general deterioration was defined by 100% of the patients as a “crisis.” They believed it to be a transitory crisis which would end once confinement and the pandemic ended. Despite distinct marital status, none of participants were living alone during the strict confinement, nor confronted it in their own room but in a house or apartment. It can be seen that 79.17% of the patients described a worsening in the relationship with their partner, and 87.50% mentioned a deterioration in their relationships with the community. All of the patients showed emotional deterioration. In 87.50% of the cases, patients believed the cause to be the situation of confinement; the same percentage alluded to overstimulation from sounds both within the family and in the neighborhood. Having to live with move movement and the resulting sounds, both in the morning and at night, was cited by 83.33% of the participants. All of the patients described having experienced increased hypersensitivity in general, including to sounds and movements. Feeling more hypersensitive and hyper-vigilant at bedtime was mentioned by 83.33%, due to noises produced in the neighborhood, or by family members and pets: both in their own and neighbors' homes. Fear of being infected by COVID-19 was cited by 62.50% and the same percentage said that their ailments and discomfort increased, due to the change in routine and lack of physical exercise, as other authors have also observed (50).

**Table 2:** Self-reporting on confinement: Transitory crises, deterioration of relationships and emotions, and causes.

	**Total**	**Women**	**Men**
	**(%)**	**(*n* = 16)**	**(*n* = 8)**
Transitory crisis	100.0	16	8
Relationships			
With the partner worsened	79.27	14	5
With a neighbor worsened	87.5	15	6
With the family worsened	25.0	4	2
Emotional state			
Worsened	100.0	16	8
Symptoms that I already had have worsened	41.7	8	2
New symptoms have appeared	12.5	2	1
Causes			
Increased general hypersensitivity	100.0	16	8
Increased hypersensitivity to sounds and movements	100.0	16	8
Overstimulation caused by sounds in the family and the neighborhood	87.5	14	7
The confinement situation	87.5	14	7
Movements and multisensory stimuli during the day and at night	83.3	14	6
Increased hypersensitivity due to sleep disorders, caused by neighborhood sounds	83.3	14	6
Increased hypersensitivity due to sleep disorders, caused by family and pet sounds	83.3	14	6
Increased hypersensitivity due to changes in routine and a lack of physical exercise	62.5	10	5
Fear of getting infected with COVID-19 and dying	62.5	10	5

In the following paragraphs, we provide a detailed overview of the patients' open-ended comments about their strategies for coping with stress, and other reflections on their fears and the confinement period. The patients recognized that the frequency with which they experienced the variables related to hyper-vigilance and hyper-sensibility, described in Table 2, increased as the days of confinement passed. They claimed to have greater resilience to triggering stimuli (movements and sounds), thanks to having previously completed training in self-control techniques, including breathing, visualization, and relaxation exercises. Most of them agreed that being well-practiced in these techniques could improve their capacity for self-control and their ability to manage triggering stimuli and/or adverse situations.

They also placed great value on having scheduled activities that they had to carry out on a daily basis: the medical psychology center had asked them to perform the activities, and monitored this throughout the confinement period. These activities had been designed on a completely individual basis, according to how the patients' condition progressed.

To tackle information overload regarding COVID-19, deaths, and the risk of contagion, measures were taken such as limiting information and performing therapeutic writing exercises to control fears and anxiety. Other measures taken to address this and other issues, were performing daily physical exercise, adjusting chronobiological factors – sleep-wake cycles and meal times – and practicing various self-control exercises.

The patients told researchers how forced confinement was implemented in an excessively sudden manner, which made it difficult for them to prepare therapeutic strategies that would enable them to deal with the situation, and left them unprepared on a psychological, physical and organizational level. They considered these factors to have aggravated the effects of confinement and made their subsequent recovery less effective.

All the strategies employed by misophonic patients during this period could be useful for anyone experiencing a situation of confinement. However, this should not detract from the suitability or effectiveness of the intervention, since we must bear in mind that the threshold stress levels of a person with misophonia are those experienced by any other person in a confinement situation. Living with constant auditory stimuli or disturbing sounds, without being able to control, avoid, run away from or manage them is to live in a state of permanent stress. In addition, misophonic patients receive a lack of empathy usually due to ignorance – from his or her family, society, and even the health system.

Participants also described how, once they had entered a stressful situation or felt unstable, they were unable to return to a state of normality by performing self-control exercises, especially if triggering stimuli occurred during the day and night. They thought that practicing self-control exercises could be somewhat more effective after “venting.” This was achieved through behavior such as getting angry, yelling, singing, running, hitting an object, playing music, or putting on any device at a very high volume. After this “shock,” some patients found it helpful to: isolate themselves from others (if they were able); do self-control exercises; put herbal heat packs (or pads) on their ears, covering both ears entirely; listen to relaxing music; do gentle physical exercise, or have a very hot shower or bath. These observations, made during the semi-structured interview, tally with the notes and statements that the patients made during the post-confinement HRV assessment.

## Discussion

This study examined the secondary impact of situations related to COVID-19 and the strict confinement measures implemented in Spain upon the fears, relationships, and emotional states of a group of people with misophonia, who were regularly attending a medical psychology center in Barcelona. Most importantly, it explored whether HRV was useful as a physiological indicator of their distress, this also being compared to their basal levels, recorded during the preceding year. The difference between the number of male and female subjects in the sample, observed in our preceding work (51), was also confirmed. Our results show that the three-month confinement period caused these patients general emotional distress and a significant increase in physiological arousal. Triggering auditory stimuli caused aversive responses, lowering HRV scores and thus increasing the stress level and heart rate, with a sustained loss of cardiac coherence. More importantly, not only real but imagined or remembered stimuli were able to cause these effects, despite the fact that there was no audio reproduction.

Some interesting results were obtained for the relaxation or rest stage, in which patients spent 10 min performing a relaxation exercise that they practiced every day at home. In the first stage, which established the basal level, and in the second assessment, the HRV scores were within the normal range of > 50. These results show that the patients, at that moment, presented a normal level of HRV in the absence of stress. However, during the post-confinement assessments, there was a drop in HRV at the resting stage, which was maintained in all three assessments. In the first post-confinement assessment, where the patients performed 10 min of relaxation and had not yet been exposed to any sound, they did not manage to raise their HRV level to a normal state. The same occurred in the next two relaxation stages, within the same post-confinement assessment period. These results suggest that the patients had been experiencing a stressful situation during confinement to which they were not exposed at the time of the previous assessments (basal level and second assessment), as they themselves confirmed in the interview carried out once the study was completed, after confinement had ended.

It can be seen that patients' evaluations of auditory stimuli in the basal-level assessment were somewhat higher than their evaluations of the same stimuli during the second assessment. In other words, the patients presented a higher level of stress in the second assessment than in the basal level. The same was true for the results of the first of the post-confinement assessments, in comparison to the previous assessments (second assessment and the basal level). It can be inferred from this that the results obtained in these post-confinement assessments are due to the alertness or hypervigilance that the patient was experiencing confinement. While the patients had not defined the auditory stimuli presented to them in the basal level assessment as triggering sounds, the participants were aware of the procedure they were about to go through, but not the type of sounds they would be exposed to. The patients lived in a state of hypervigilance resulting from the constant auditory stimuli that were present during confinement, which they experienced as a threat and therefore caused them stress (52, 53). LeDoux posed the hypothesis of increased activity in the amygdala (54, 55) and the superior temporal cortex being related to maintaining greater attention to sounds (56, 57).

Another factor that supports this interpretation is the progressive decrease in scores in all the assessments, recovery not having been possible, despite patients having taken 5 min of rest and carrying out a relaxation exercise that they knew and had practiced before. Added to this was the cumulative effect that occurred in all the assessments, especially at the post-confinement stage. According to Schröder et al. (58), repeated exposure to triggering stimuli may increase the severity of reactions. The data in this study demonstrate this cumulative effect, caused by the succession of sounds, and show that the time provided for recovery was not enough. The participants themselves made the case for another solution: most of them mentioned a need to get out of the room, run, move, yell, scream, talk or do any activity that involved physical movement and verbal expression. The relaxation exercise, after exposure to sound, was an impediment rather than a help. However, before any auditory exposure, relaxation exercises did reduce the effect of triggering sounds or prevent these effects from occurring as quickly.

A decrease in the HRV score for the second of the post-confinement assessments was to be expected, since this one exposed the patient to the triggering auditory stimuli that he or she had predetermined before confinement and during the second assessment in the program. However, it is worth noting how these scores compare to the following assessment, the third in the post-confinement stage. In this last assessment (post-confinement 3), the patients were asked to remember or imagine the triggering sounds they had heard in the previous assessment (post-confinement 2). Therefore, in this last assessment, the sounds were not audible. Comparing both groups, a recovery in HRV scores can be observed in the third post-confinement assessment. The patients therefore experienced less stress in this final assessment. The conclusion we may draw is that triggering sounds, even if they are not those heard by patients in their daily lives, having been extracted from a database, generate greater distress than the imagined sounds. Although more in-depth study is required, considering this observation is of special interest for misophonia treatment.

In both the second assessment and the post-confinement stage, there was a negative relationship between HRV scores and the severity of misophonia. The more severe the degree of misophonia, the lower the HRV score. Moreover, this occurred during periods of relaxation and when exposed to auditory stimuli, which confirms that when a patient has a higher level of misophonia, both hypervigilance and stress levels are more pronounced. Here it is important to clarify that misophonia was assessed using the Amsterdam Misophonia Scale [A-MISO-S, (41)] and the ‘levels of misophonia' used in the present work were defined by the authors for the only purpose to facilitate analysis in categories.

As expected, in the semi-structured interview, all participants self-reported a deterioration in their emotional status and experiencing a transitory crisis. They all attributed these changes to a general sense of hypersensitivity, as well as hypersensitivity. Feeling particularly sensitive, due to the change in their routines and a lack of physical activity, were also cited as causes of their worsening psychological health. The fear of getting infected with COVID-19 and dying was also mentioned by 10 out of 16 women, and five out of eight men. According to a previous article published by the authors (51), once the lockdown was finished, most misophonia patients attending the medical center reported to practicing self-confinement, their main reason for doing so being the fear of being infected with or dying from COVID-19.

Conflicts with participants' partners, and especially with their neighbors, were caused by the noise they made throughout the day and part of the night. The long, strict confinement period made this a recurrent situation that was impossible to avoid. This caused a crisis on the personal and interpersonal levels, as it affected their relationships with their neighbors and partners. Patients described the crisis as transient because they directly related it to the situation of confinement and the fact that it was difficult to manage the problems. However, the lack of empathy for people with misophonia – sometimes due to ignorance, but mostly due to a lack of consideration – was another aggravating factor in this crisis. A recent study (12) showed there to be neuronal correlates of misophonia that are triggered by audiovisual stimuli, resulting in a conditioned response involving anger and increased physical arousal. Thus, increased activation of the right insula, anterior cingulate cortex and superior temporal cortex was observed in their patients. According to the authors, given that these areas are involved in the moral evaluation of stimuli (59, 60), patients with misophonia may have perceived these stimuli as personal harassment, triggering subsequent anger. With respect to our study, in the ongoing COVID-19 pandemic, fear of the disease and death, as well as present and future uncertainty (this paper and 32) occur in contrasting situations: being exposed to inescapable sounds inside the home, and the silence of empty cities and streets. The sensorimotor gating process may have come up against an unprecedented neurophysiological sensorial imbalance that, in turn, may have enhanced individuals' physiological arousal, heightening their perception of the sounds around them and their interpretation of these sounds as aversive stimuli.

In the context of an extreme situation such as confinement, the most desirable strategy would be to take preventive measures, so that people with misophonia do not develop behaviors and emotional states that are undesirable, both for themselves and the people with whom they live. Based on results of this study, we have produced a series of guidelines and recommendations, which are detailed below.

[1] Awareness needs to be raised about the problem and what its triggers are.
[2] The awareness of family members or the people with whom patients live also needs to be raised.
[3] Prior to being exposed to any extreme situation, such as confinement, the person suffering from misophonia should have learned and internalized relaxation exercises, self-control techniques, and controlled breathing. Other interventions that can help include:
[4] Fitting bedtime and the time for restorative sleep to the circadian rhythm. Avoiding long naps and heavy meals close to bedtime. Alcoholic or caffeinated drinks, or any other stimulants, should also be avoided.
[5] Doing physical exercise daily, as well as having designated objectives to meet and scheduled activities to perform.
[6] When sounds are unavoidable, it is helpful to have a repertoire of prepared strategies to help muffle them, such as: white noise, listening to music on headphones, noise-canceling headphones. In these situations, and to relax, it is effective to place a soft heat pack on the auditory pinna.

## Conclusions

The extreme, unprecedented situations (the fear/risk of death and confinement) caused by the current COVID-19 pandemic have threatened to worsen the symptoms of misophonia sufferers and, consequently, their physical and psychological health. The results of this study, obtained from a sample of people with moderate to extreme misophonia who underwent the strict confinement imposed in Spain, confirm the usefulness of home-monitored HVR to identify a worsening responsiveness to auditory triggers, with respect to two previous basal periods. The loss of cardiac coherence was greater for patients with more severe misophonia. Additionally, imagined or evoked triggering sounds, as well as real ones, resulted in a loss of cardiac coherence. At the same time, the deterioration in patients' interpersonal relationships, especially with partners and neighbors, requires more initiatives at a clinical level but also at a societal level. Finally, participants handled their worsening situations with several coping strategies and by considering the crisis a transitory one that was associated with the severe conditions of confinement. Raising awareness about this disorder would help address the current lack of health and social care and hopefully prevent the impact of this shortfall, currently evidenced in a deterioration in social and affective relationships.

## Data Availability Statement

The raw data supporting the conclusions of this article will be made available by the authors, without undue reservation.

## Ethics Statement

The studies involving human participants were reviewed and approved by L'Alfatier Medical Center. The patients/participants provided their written informed consent to participate in this study.

## Author Contributions

AF-T: interviews, data collection and analysis, and manuscript draft. All authors contributed equally to the concept, design, and scientific discussion, as well as manuscript writing and approval.

## Conflict of Interest

The authors declare that the research was conducted in the absence of any commercial or financial relationships that could be construed as a potential conflict of interest.
