# Supplementary material for: Sounds of Silence in Times of COVID-19: Distress and Loss of Cardiac Coherence in People With Misophonia Caused by Real, Imagined or Evoked Triggering Sounds
Source: Front Psychiatry. 2021 Jun 30;12:638949. doi: 10.3389/fpsyt.2021.638949 (PMC8278014; doi:10.3389/fpsyt.2021.638949)
Supplement: Supplementary file 1 [file Data_Sheet_1.PDF]

Supplementary Data

Misophonia level - Medians and quartiles

Moderate M+ (Level 2)	n	Median	Percentile 25	50	75
**Retest**					
RT Relax 10 min	10	84,3000	81,2500	84,3000	89,5000
RT Food/mouth neutral sound 1	10	72,7000	70,9250	72,7000	78,5000
RT Nasal neutral sound 1	10	69,7500	67,3750	69,7500	74,8250
RT Another neutral sound 1	10	60,2000	54,1500	60,2000	63,7500
**Confinement – 1rst evaluation**					
CO 1. Rest 10 min	10	53,4000	50,4750	53,4000	66,2750
CO 1. Food/mouth neutral sound 1	10	45,6500	43,5750	45,6500	49,4000
CO 1. Nasal neutral sound 1	10	42,4000	41,2000	42,4000	45,9250
CO 1. Another neutral sound 1	10	40,9000	40,0500	40,9000	43,9250
**Confinement – 2nd evaluation**					
CO 2. Relax 10 min	10	49,0000	48,2750	49,0000	49,5000
CO 2. Food/mouth trigger sound A	10	43,4000	38,5000	43,4000	45,3250
CO 2. Nasal trigger sound A	10	40,8000	34,5500	40,8000	42,3750
CO 2. Another trigger sound A	10	39,3000	28,9500	39,3000	40,5500
**Confinement – 3rd evaluation**					
CO 3. Relax 10 min	10	48,6500	44,7250	48,6500	49,1000
CO 3. Food/mouth imagined sound A	10	45,3000	39,8000	45,3000	47,8250
CO 3. Nasal imagined trigger sound A	10	43,8500	38,7500	43,8500	45,2250
CO 3. Another imagined trigger A	10	37,8500	34,1500	37,8500	43,2250

Severe M+ (Level 3)	n	Median	Percentile 25	50	75
**Retest**	10	55,3500	50,9500	55,3500	60,3250
RT Relax 10 min	10	45,9000	43,9750	45,9000	47,5250
RT Food/mouth neutral sound 1	10	43,7000	40,6000	43,7000	45,1750
RT Nasal neutral sound 1	10	39,7500	34,1250	39,7500	43,3250
RT Another neutral sound 1	0				
**Confinement – 1rst evaluation**	10	36,4500	29,6000	36,4500	42,4000
CO 1. Rest 10 min	10	33,3500	24,2250	33,3500	38,6750
CO 1. Food/mouth neutral sound 1	10	29,2500	19,3000	29,2500	35,5750
CO 1. Nasal neutral sound 1	10	28,3000	15,9250	28,3000	30,5500
CO 1. Another neutral sound 1	0				
**Confinement – 2nd evaluation**	10	34,3500	27,7750	34,3500	40,2500
CO 2. Relax 10 min	10	33,3000	22,1000	33,3000	34,9500
CO 2. Food/mouth trigger sound A	10	29,8000	17,7000	29,8000	32,6500
CO 2. Nasal trigger sound A	10	25,3000	14,5500	25,3000	29,7250
CO 2. Another trigger sound A	0				
**Confinement – 3rd evaluation**	10	34,7500	25,3250	34,7500	38,1250
CO 3. Relax 10 min	10	33,5000	22,5000	33,5000	35,9000
CO 3. Food/mouth imagined sound A	10	33,0500	20,1250	33,0500	35,3750
CO 3. Nasal imagined trigger sound A	10	30,8500	16,0500	30,8500	34,4750

Very severe M+ (Level 4)	n	Median	Percentile 25	50	75
**Retest**					
RT Relax 10 min	4	31,0500	29,2250	31,0500	38,5000
RT Food/mouth neutral sound 1	4	20,4000	15,2250	20,4000	27,9000
RT Nasal neutral sound 1	4	13,6000	9,5000	13,6000	15,0000
RT Another neutral sound 1	4	8,5500	7,7250	8,5500	9,1500
**Confinement – 1rst evaluation**					
CO 1. Rest 10 min	4	10,6000	6,5000	10,6000	12,3000
CO 1. Food/mouth neutral sound 1	4	7,5500	4,0500	7,5500	9,7000
CO 1. Nasal neutral sound 1	4	5,7000	4,5000	5,7000	6,0750
CO 1. Another neutral sound 1	4	4,4500	3,5250	4,4500	5,3750
**Confinement – 2nd evaluation**					
CO 2. Relax 10 min	4	9,7000	5,9250	9,7000	11,0750
CO 2. Food/mouth trigger sound A	4	5,4500	2,7500	5,4500	8,3750
CO 2. Nasal trigger sound A	4	3,8000	2,4750	3,8000	4,0750
CO 2. Another trigger sound A	4	2,8000	2,4500	2,8000	3,1500
**Confinement – 3rd evaluation**	4	8,4000	5,4750	8,4000	10,2000
CO 3. Relax 10 min	4	6,6000	3,8750	6,6000	8,9500
CO 3. Food/mouth imagined sound A	4	6,4500	3,8500	6,4500	7,4750
CO 3. Nasal imagined trigger sound A	4	3,6000	2,4250	3,6000	4,9250

Confinement – HRV vs Baseline	Kruskal-Wallis	gl	P=
CO 1. Food/mouth neutral sound 1	8,000	1	.005
CO 1. Nasal neutral sound 1	8,018	1	.005
CO 1. Another neutral sound 1	8,018	1	.005
CO 2. Food/mouth trigger sound A	8,000	1	.005
CO 2. Nasal trigger sound A	8,000	1	.005
CO 2. Another trigger sound A	8,000	1	.005
CO 3. Food/mouth imagined sound A	8,018	1	.005
CO 3. Nasal imagined trigger sound A	8,000	1	.005
CO 3. Another imagined trigger A	8,000	1	.005
